# Assessment of Potential Sources of Variability on the Analytical Performance of a Circulating Tumor DNA Minimal Residual Disease Test for B‐Cell Lymphomas

**DOI:** 10.1002/jcla.70258

**Published:** 2026-05-06

**Authors:** Nina Klimova, Alanna Roff, Justine McCutcheon, Alex Aheimer, Sandra L. Close, Richard D. Hockett, Stephanie Meek, Laura Hyland

**Affiliations:** ^1^ Foresight Diagnostics, Inc. Boulder Colorado USA

**Keywords:** CLARITY, ctDNA, MRD, PhasED‐Seq, residual disease

## Abstract

**Aims:**

To evaluate the robustness of circulating tumor DNA minimal residual disease (ctDNA‐MRD) test results to common factors that could impact performance.

**Methods:**

DNA from three sample types is required: somatic, germline, and MRD monitoring. Different sample sources were evaluated for germline [whole blood, peripheral blood mononuclear cells (PBMCs)] and somatic (pre‐treatment plasma, tumor) samples. Extraction methods, common interfering substances, and DNA input mass were evaluated for impact on MRD determination. Each comparison was performed in isolation. Overall test variability was also evaluated.

**Results:**

Whole blood and PBMCs were acceptable germline sample types, with minimal differences in identification of somatic variants. Both pre‐treatment plasma and tumor were acceptable somatic sample types, achieving the same 95% hit rate of three mutant molecules. Agreement between results from different extraction methods and in the presence of interfering substances was 100%. Positive percent agreement (PPA) between results from DNA input masses above and below the test upper and lower limits decreased, though PPA was 100% between 4 ng and the lower limit (5 ng). Variance in test performance fell within acceptable ranges (coefficient of variation 4.95%–10.33%).

**Conclusions:**

Major potential sources of variation and interference did not significantly impact ctDNA‐MRD test performance.

## Graphical abstract

Laboratory tests must exhibit robustness to common sources of test variability, particularly for tests with the potential to inform treatment decisions. This study presents data that shows that a phased variant enrichment and detection sequencing (PhasED‐Seq)‐based circulating tumor DNA minimal residual disease (ctDNA‐MRD) test is robust, with results independent of sample type, interfering substances, extraction method, and DNA input mass. Combined with previous clinical studies, this further supports the clinical use of the ctDNA‐MRD test for patients with B‐cell lymphomas.

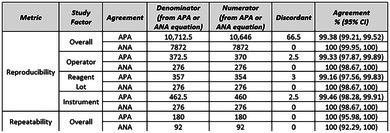

## 1 Introduction

Diffuse large B‐cell lymphoma (DLBCL) is the most common subtype of non‐Hodgkin lymphoma in developed countries [1]. Standard frontline therapy for patients with DLBCL includes treatment with R‐CHOP (rituximab, cyclophosphamide, doxorubicin, vincristine, and prednisone) or similar therapies, with or without radiation [2]. Despite alterations to conventional frontline therapies with the goal of increasing efficacy, relapse after treatment or patients becoming refractory to these frontline therapies is not uncommon [3]. Response criteria currently rely primarily on functional radiographic tools such as positron emission tomography/computed tomography (PET/CT), which are limited in regard to sensitivity and specificity, resulting in false‐positive and false‐negative rates that are higher than would be clinically ideal [4]. Better tools for measuring residual disease and response to therapy are desired to improve patient outcomes.

To address this clinical need, a non‐invasive circulating tumor DNA‐based minimal residual disease (ctDNA‐MRD) test has been developed. This test is based on phased variant enrichment and detection sequencing (PhasED‐Seq) which leverages phased variants (PVs) to improve the sensitivity of ctDNA detection compared to other single nucleotide variant (SNV) based approaches [5]. Recently, the National Comprehensive Cancer Network (NCCN) has recognized the utility of such tests and now recommends using an ultra‐sensitive ctDNA‐MRD test to adjudicate positive PET results to minimize treating false‐positive patients [6]. Additionally, the results of the ctDNA‐MRD test may also be used to adjudicate negative PET results, as false‐negative PET scans have been shown to occur [7], though adoption into clinical guidelines for this use is still pending. While the analytical and clinical validity of the ctDNA‐MRD test for B‐cell lymphomas has been investigated [8, 9, 10, 11, 12, 13], test robustness has not yet been established. Understanding the impact of common sources of variability is critical to understanding the reliability of the MRD determination results. Here, we investigate factors such as sample type, DNA extraction methods, interfering substances, and input mass, all of which may impact the performance of the ctDNA‐MRD test.

## 2 Methods

### 2.1 ctDNA‐MRD Testing

The Foresight CLARITY MRD test (Foresight Diagnostics Inc.) has been previously described [8]. Briefly, this centralized, single site ctDNA‐MRD test utilizes pre‐treatment plasma or tumor tissue (somatic), germline DNA (gDNA; normal), and an MRD monitoring sample. DNA is extracted and sequenced using a fixed hybrid capture panel which enriches for specific genomic regions known to contain PVs common in B‐cell lymphomas [11]. This fixed hybrid capture panel was designed without genomic regions where clonal hematopoiesis of indeterminate potential (CHIP) variants commonly occur. These CHIP mutations also occur as SNVs [14], rather than PVs, which means that they are not detected as part of PhasED‐Seq testing. Additionally, the use of germline subtraction reduces the risk of CHIP variant inclusion in the construction of patient‐specific PV lists. This was done to avoid any impact from CHIP on MRD test performance. Using the somatic and normal gDNA samples, a tumor‐specific PV list is generated by identifying PVs that are present only in the somatic but not in the normal samples (i.e., subtracting the PVs in the normal samples from those identified in the somatic sample). MRD status is then determined by applying this tumor‐specific PV list to the MRD monitoring sample.

Unless otherwise specified below, blood was collected using Streck cell‐free DNA (cfDNA) blood collection tubes (BCTs). cfDNA was isolated from plasma using either the QIAsymphony DSP Circulating DNA kit (Qiagen, Hilden, Germany) on the automated QIAsymphony system or the Mag‐Bind cfDNA kit (Omega Bio‐tek, Norcross, GA) on the automated Hamilton platform (Hamilton Company, Renok, NV). Double‐stranded DNA quantification was performed using a Qubit Fluorometer with the Qubit dsDNA High Sensitivity Assay Kit (Invitrogen, Waltham, MA) or the Quant‐iT PicoGreen dsDNA Assay Kit (ThermoFisher Scientific, Waltham, MA). Genomic DNA was isolated using either the QIAsymphony DSP DNA Mini Kit (Qiagen) on the automated QIAsymphony system or the Mag‐Bind Blood DNA HDQ Kit (Omega Bio‐tek) on the automated KingFisher platform (ThermoFisher Scientific, Waltham, MA), sheared by sonication, and quantified using the Qubit dsDNA Broad Range Assay Kit (Invitrogen) or the Quant‐It PicoGreen Assay. Library preparation, hybrid capture target enrichment, and sequencing by synthesis were performed according to optimized workflows under standard operating procedures, as previously described [8].

### 2.2 Robustness Studies

For convenience, a table summarizing sample numbers, replicate counts, and experimental conditions for all of the following studies is provided in the data supplement (Table S1). Statistical analyses were performed using R version 4.3.2. Publicly available software used for the MRD testing process was previously reported [8].

#### 2.2.1 Sample Source Comparisons: Germline DNA

Different sources of germline DNA (gDNA) were compared for use as the normal sample for the ctDNA‐MRD test. Whole blood (WB) from 15 self‐reported cancer‐free donors was obtained from a commercial vendor (Discovery Life Sciences). Blood was collected in K2‐EDTA blood collection tubes (BCTs) and shipped to Foresight Diagnostics Inc. (Boulder, CO) for processing. Upon receipt, gDNA was extracted in duplicate from each sample, both directly from WB and from peripheral blood mononuclear cells (PBMCs) isolated from WB. Fragmentation of gDNA was performed by sonication to approximately 300 base pairs (bp) and library preparation was performed using 80 ng input mass for a total of four samples from each donor (two each from PBMCs and WB), resulting in a total of 60 libraries. Library samples were subsequently captured for target enrichment and sequenced using a NovaSeq X Plus.

PV lists were generated for each donor using all possible combinations of the WB‐ and PBMC‐derived gDNA replicates set as either the somatic or normal sample (Table S2). As gDNA, isolated from either WB or PBMCs, was used for both the normal and somatic sample in this comparison, it was expected that PV lists would contain zero PVs as PV lists are generated by subtracting any PVs identified in the normal sample from those identified in the somatic sample. For each donor, any PV lists where PVs were identified were reviewed for differences in PVs detected across replicates and gDNA sample sources. Any donors with non‐empty PV lists were also analyzed by a chi‐square test to determine whether the population of PVs identified from PBMCs was significantly different from the population of PVs identified from WB.

#### 2.2.2 Sample Source Comparisons: Somatic

The limit of detection (LoD) of the ctDNA‐MRD test using either pre‐treatment plasma or tumor tissue as the somatic sample type was assessed. To determine the LoD of the test (95% detection rate per CLSI EP17‐A2), a limited dilution series of a DLBCL clinical‐contrived sample was prepared at six targeted phased variant allele fraction (PVAF) levels (7.00E‐06, 3.50E‐06, 1.75E‐06, 8.75E‐07, 4.38E‐07, and 2.19E‐07; 10 replicates per dilution level). Clinical‐contrived sample replicates were created by combining cfDNA from four DLBCL patient samples and diluting the mixture into background cfDNA from healthy donor plasma. The targeted PVAF levels, used to determine the LoD, are based on the amount of informative molecules detected for the clinical‐contrived sample. The PV list can be generated from two somatic sample types: pre‐treatment plasma or tumor tissue. To assess whether the somatic sample type has an impact on LoD, the clinical‐contrived sample had two PV lists derived, one from pre‐treatment plasma and one from pre‐treatment tumor tissue. The LoD95 was previously established for this test by Klimova et al. [8]. Based on the test following Poisson distribution, we would expect a LoD95 of approximately three mutant molecules when the MRD calling threshold is 0.

The plasma‐ and tumor‐derived PV lists were used for detection rates. The detection rate for each PVAF level was calculated by adding the number of MRD positive calls and dividing by the total number of replicates tested. A probit model was used to compute the number of mutant molecules and the PVAF corresponding to a detection rate of 95%. Binary detection results were regressed against corrected targeted PVAF or mutant molecules. Corrected PVAF levels were calculated using the highest observed PVAF level as a reference point and remaining dilution levels were then calculated based on the dilution ratios used in the series. Estimated model parameters (−0.92 and 0.62) were used to algebraically solve the regression equation to find the LoD value which corresponded to the modeled 95% probability of detection. Probit model fit was determined to be acceptable by evaluating with a statistical goodness of fit test. Detection rates, probit models for PVAF, and probit models for mutant molecules detected determined using plasma‐ and tissue‐based PV lists were compared.

#### 2.2.3 Interfering Substances

The impact of common endogenous interfering substances on the ctDNA‐MRD test was assessed. These interfering substances are commonly found in the plasma and blood and include conjugated bilirubin, hemoglobin, and triglycerides. For the purpose of these analytical studies, the interferant concentrations tested were based on CLSI EP37 [15]. Plasma sample types consisted of contrived healthy donor plasma background and sonically sheared B‐cell lymphoma cell line DB or NU‐DUL‐1 (ATCC, Manassas, VA) at two PVAF levels (0.02% and 0.01%). A total of five unique sample types were generated: Two cell line‐contrived samples at two PVAF levels each and one uncontrived healthy donor sample. Interfering substances were individually spiked into each sample type in duplicate.

PV lists were generated from cell lines. cfDNA was extracted and each extract had library input normalized to 15 ng. Library preparation, hybrid capture, and sequencing were performed and analyzed for impact of interfering substances using positive percent agreement (PPA), defined as the probability of a MRD‐positive result occurring in known MRD‐positive samples.

Agreement was also evaluated at low input masses: 7.5 and 5 ng (the lower limit of the test). PPA was defined here as the probability of a MRD positive result occurring in known MRD positive samples. The number of informative molecules (i.e., the number of cfDNA molecules spanning the location of a tumor‐specific PV) for these lower input masses were similarly reduced, with the 7.5 ng input mass having half as many informative molecules as the 15 ng input mass and the 5 ng input mass having a third as many. The expected number of mutant molecules were simulated at the lower input mass levels using Monte Carlo sampling with 100,000 iterations from a binominal distribution based on the observed PVAF and informative molecule counts. The number of informative molecules for these lower input masses were reduced, with the 7.5 ng input mass having half as many informative molecules as the 15 ng input mass and the 5 ng input mass having a third as many. Agreement was evaluated for 7.5 and 5 ng as well.

#### 2.2.4 Extraction Method Comparison

To evaluate whether the cfDNA extraction method impacts ctDNA‐MRD test performance, plasma was collected from 14 healthy donors. Donors were age matched to the intended use population of the ctDNA‐MRD test (≥ 45 years of age). Samples from 10 of the healthy donors had DLBCL donor cfDNA that was MRD positive added, and two donors had DLBCL donor cfDNA that was MRD negative added, thus creating clinical‐contrived samples with expected MRD calls. The remaining two donors were not altered using added DLBCL donor cfDNA (healthy donors).

A total of 44 libraries were prepared: 28 libraries for analyses, 4 control libraries (2 positive and 2 negative from each kit), and 12 libraries that underwent plasma pool characterization to be used as the normal sample. Of the 28 libraries analyzed, 14 matched replicates were processed with both the QIAsymphony DSP Circulating DNA Kit (Qiagen) and the Mag‐Bind cfDNA Kit (Omega). cfDNA fragment traces (50–450 bp) of the extracted cfDNA were compared between the two extraction methods. PPA, negative percent agreement (NPA), and overall percent agreement (OPA) were calculated. Specificity was evaluated by interrogating two additional healthy donors against the PV lists generated, with an expected result of MRD ABSENT for these donors.

#### 2.2.5 DNA Input Mass

Robustness of the ctDNA‐MRD test's DNA input mass was evaluated by testing a range of DNA input masses (5 input mass levels: 2.5, 4, 5, 120, and 144 ng) using DLBCL clinical‐contrived samples near the LoD of the test. The lower test input masses of 2.5 and 4 ng (50% and 20% lower than the reference minimum) were compared to the minimum reference input mass of 5 ng. The higher test input mass of 144 ng (20% higher than the reference maximum) was compared to the maximum reference input mass of 120 ng. Four replicates of each of the clinical‐contrived samples were tested across the range of DNA input masses. A total of 60 libraries were prepared. MRD call concordance between the test and reference conditions was evaluated using PPA; the expected MRD result for all samples was MRD PRESENT. Mutant molecules per sample and input mass level were also evaluated.

#### 2.2.6 Reproducibility and Repeatability

DLBCL clinical‐contrived samples [3 MRD positive samples and 2 MRD negative samples collected following first‐line immunochemotherapy (EOT)] were used to evaluate ctDNA‐MRD test reproducibility and repeatability. MRD positive sample replicates that were evaluated for reproducibility and repeatability had a range of observed PVAFs that were 0.5 to 10 times the estimated LoD at 5 and 120 ng input mass. MRD negative samples were evaluated using 5 ng input mass. Sample replicates were tested across two unique reagent lots and operators, three instruments, and across eight separate runs. Operator 1 used instrument 1, while operator 2 used instruments 2 and 3, which by nature confounds operator and instrument in variance analyses. Repeatability and reproducibility were assessed using average positive and negative agreement (APA and ANA, respectively) of MRD calls, as previously described [16]. The impact of operator, instrument, and reagent lot on test precision was also assessed.

Test variation was assessed using informative and per‐sample summary statistics were assessed: mean, standard deviation (SD), and percent of coefficient of variation (CV%). It is expected that, per‐sample, the informative molecules will stay consistent across preparation for any specific input mass, assuming a similar sequence depth. Variance component analysis (VCA) using a linear mixed effects model was performed interrogating informative molecules to determine significant sources of variance in the ctDNA‐MRD test and the relative contributions of different factors (e.g., reagent lot, operator) to the overall variability. Due to the confounded nature of operator and instrument as indicated above, the operator variance component captures most of the operator‐instrument variability as a single variance component. While operator 2's use of multiple instruments provides some separation between these factors, the limited replicates preclude estimating instrument as an independent variance component, and therefore instrument was not evaluated separately. Any remaining unexplained variability is captured in the residual variance measure.

### 2.3 Statistical Analyses

Unless otherwise specified, statistical differences between groups were assessed using chi‐square tests for categorical variables and by examining the overlap of 95% confidence intervals and boxplot distributions for continuous variables. Groups were considered significantly different when their confidence intervals or plots did not overlap or when chi‐square tests yielded *p* < 0.05.

## 3 Results

### 3.1 Sample Source Comparison: Germline DNA

Two different sample types were used to isolate the gDNA samples required for MRD testing, WB and PBMCs. Samples were sequenced at comparable depths across experiments. The median molecule depth was 4293 (range 3801–4933) for PBMCs and 4817.5 (range 3986–5304) for WB. For the interest of this comparison, this gDNA was used in various combinations (Table S2) as both the somatic and normal inputs, with the expectation that PV lists would be generated as “empty” or lacking any unique PVs that occur in only the “somatic” sample and not the “normal.” A total of 180 comparisons were generated from analysis of all 60 libraries prepared using PBMCs and WB. Across all comparisons, 96.7% (174/180) resulted in a differential PV list size of zero, which indicated that the same PVs were identified from both sample types (Table S3). Only 6 out of 180 (3.3%) combinations resulted in a non‐empty PV list. The PVs present were mostly found in a single sample, single replicate, and with few (≤ 4) mutant molecules (Table 1). These identified PVs appear due to sampling variability, as the same genomic positions were consistently observed across replicates but PVs were only observed in 1 or 2 replicates of the same sample. Additionally, chi‐square test results demonstrated that the population of PVs identified from the two sample types was not statistically significantly different for either donor (Table S4).

**TABLE 1:** Phased variants observed across sample replicates for donors with PV list discrepancies.

Donor	Phased variant	Type and replicate	Mutant molecules	Informative molecules	Aggregate PVAF
2	chr13:50057701:T:G chr13:50057772:T:G	PBMC1	1	204	0.004902
		PBMC2	2	157	0.012739
		WB1	0	197	0
		WB2	0	203	0
	chr13:50057698:T:G chr13:50057772:T:G	PBMC1	0	211	0
		PBMC2	2	160	0.012500
		WB1	0	207	0
		WB2	0	202	0
8	chr6:134495223:A:T chr6:134495235:G:C chr6:134495286:G:A	PBMC1	4	270	0.014815
		PBMC2	0	216	0
		WB1	0	305	0
		WB2	0	270	0
	chr6:134495223:A:T chr6:134495286:G:A	PBMC1	4	275	0.014545
		PBMC2	0	217	0
		WB1	0	308	0
		WB2	0	276	0
	chr6:134495235:G:C chr6:134495286:G:A	PBMC1	4	274	0.014599
		PBMC2	0	220	0
		WB1	0	316	0
		WB2	0	276	0

### 3.2 Sample Source Comparison: Somatic Plasma or Tumor

Using pre‐treatment plasma and tumor tissue from the same donors, PV lists with 10,044 and 9043 PVs were generated, respectively; the two lists shared 7685 PVs in common. Despite the plasma‐based PV list having approximately 1000 more PVs compared to the tissue‐based list, the LoDs calculated from each sample type were similar. The test algorithm employs a threshold based on the number of mutant molecules to result in an MRD‐positive determination; however, as the same threshold is applied to both sample types in this comparison, the acceptability of either tissue or plasma for the somatic sample is agnostic to the test algorithm employed.

**FIGURE 1:**
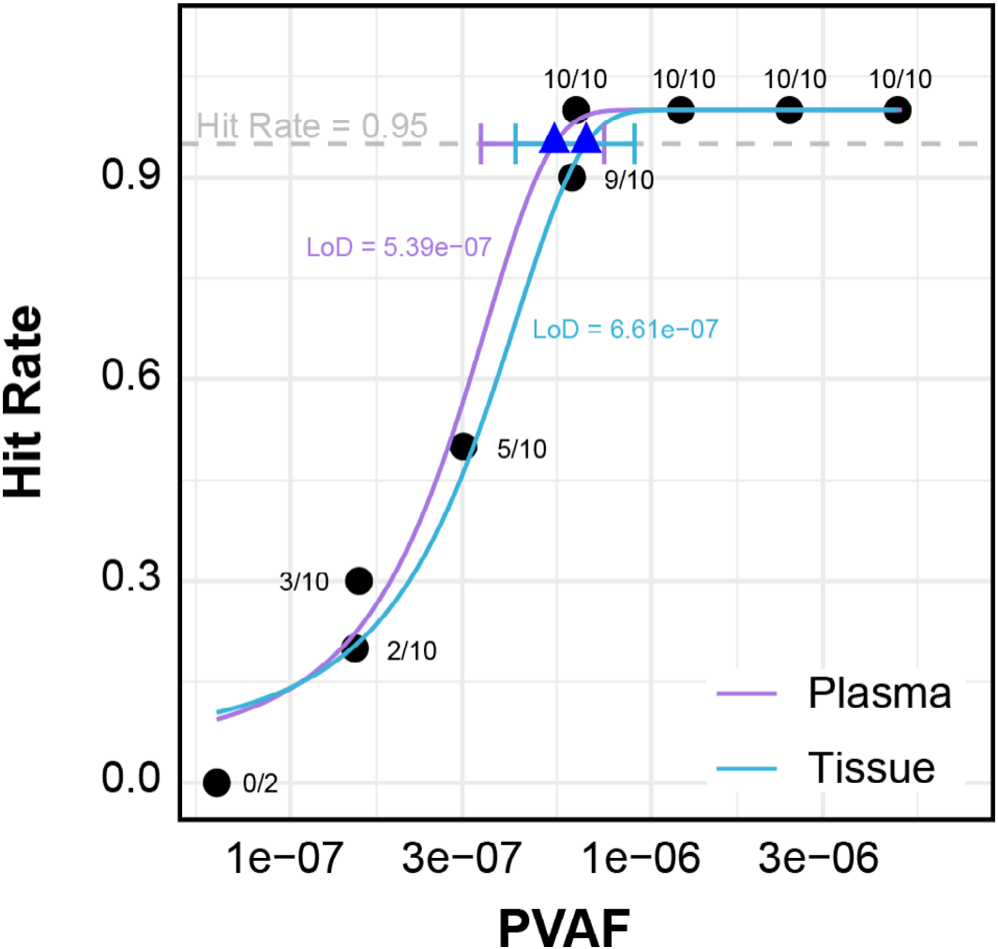
Probit modeling of limiting dilution series for the plasma‐ and tissue‐based PV lists. Phased variant allele fraction (PVAF) corresponding to a detection rate of 95% was similar (< 1 ppm).

**FIGURE 2:**
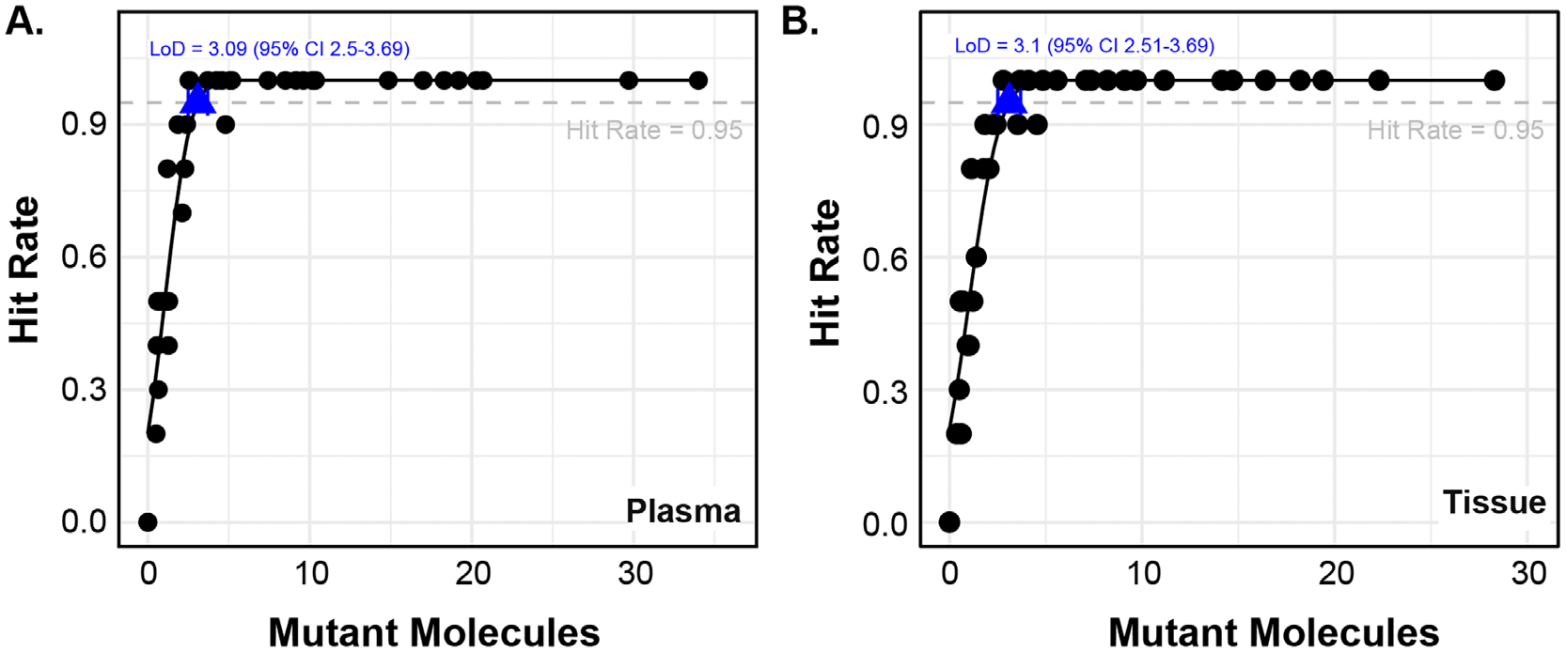
Hit rate vs. mutant molecules for (A) tissue‐based and (B) plasma‐based PV lists. Hit rates for 95% detection were nearly identical between input types (LoD 3.09 vs. 3.1).

The DLBCL clinical‐contrived sample replicates were prepared at the six targeted PVAF levels. In a dilution series ranging from approximately 5E‐06 to 1.5E‐07, PVAF was linear with the dilution of the mutant molecules and PVAF. Detection rates at each corrected PVAF level for each PV list source are shown in Table 2. The detection rates for the limiting dilution series between the two PV list sources were comparable. Two PVAF levels had discordant detection rates, but the difference was only observed in a single replicate. Based on probit modeling, the PVAF which corresponded to a detection rate of 95% was 5.39E‐07 (< 1 ppm; 95% CI 3.37E‐07 to 7.41E‐07) for the plasma‐based PV list and 6.61E‐07 (< 1 ppm; 95% CI 4.21E‐07 to 9.01E‐07) for the tissue‐based PV list (Figure 1). For both PV list sources, probit modeling converged on 3.1 mutant molecules to achieve a 95% hit rate (Figure 2). The overlapping CIs for both PVAF and mutant molecules indicate no significant difference based on sample type for the samples tested.

**TABLE 2:** Detection rates for the limiting dilution series using PV lists generated from pre‐treatment plasma or tissue.

Pre‐treatment plasma			Pre‐treatment tissue		
Corrected targeted PVAF	Part per million (ppm)	Hit rate	Corrected targeted PVAF	Part per million (ppm)	Hit rate
4.96e‐06	4.96	100%	4.83e‐06	4.83	100%
2.48e‐06	2.48	100%	2.42e‐06	2.42	100%
1.24e‐06	1.24	100%	1.21e‐06	1.21	100%
6.2e‐07	0.62	100%	6.04e‐07	0.60	90%
3.1e‐07	0.31	50%	3.02e‐07	0.30	50%
1.55e‐07	0.15	30%	1.51e‐07	0.15	20%

*Note:* Input mass was 120 ng. Hit rate was calculated as the number of MRD PRESENT calls out of 10 total.

### 3.3 Interfering Substances

**FIGURE 3:**
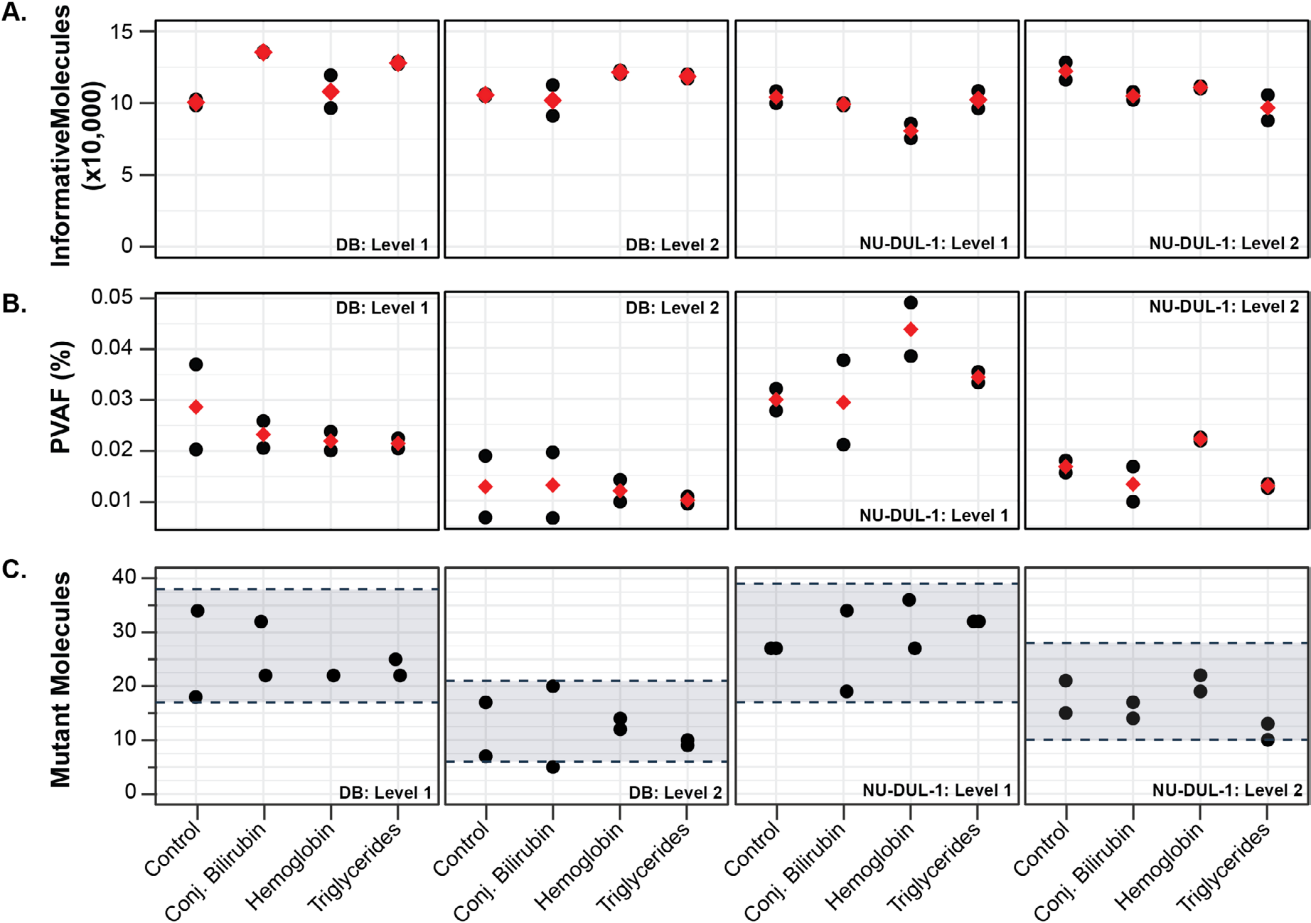
Impact of interfering substances on ctDNA‐MRD test using B‐cell lymphoma cell lines. For each interfering substance, 2 cell lines were used with two different PVAF target levels. (A) Informative molecules, (B) PVAF, and (C) mutant molecules were all evaluated. The grey box in the mutant molecules section indicates the mutant molecule Poisson distribution of control condition. Results for informative molecules, PVAF, and mutant molecules were comparable between the control and in the presence of interferents.

Overall, high levels of endogenous interfering substances did not impact the performance of the ctDNA‐MRD test regarding MRD status for known MRD positive (B‐cell lymphoma cell lines) and negative (healthy donor) samples (PPA 100%, NPA 100%, OPA 100%; Table S5). When lower input masses (7.5 and 5 ng) were evaluated, the lowest PPAs were observed in the 5 ng input mass samples, but still ranged from 97% to 99.5% across all interfering substance conditions (Table S6). The number of informative molecules, mutant molecules, and the PVAF were similar across libraries prepared, for all interfering substances tested (Figure 3, Figure S1).

### 3.4 Extraction Method Comparison

Libraries prepared from both extraction kits performed comparably, with expected variation due to random sampling and use of different instrumentation. PPA was 100% (95% CI 69.15%, 100%), NPA was 100% (95% CI 79.41%, 100%), and OPA was 100% (95% CI 86.77%, 100%). The median absolute plasma cfDNA yield was 2.91 ng/mL (minimum 2.91 ng/mL, maximum 9.55 ng/mL) for the Hamilton Omega Mag‐Bind cfDNA Kit and 4.81 ng/mL (minimum 1.29 ng/mL, maximum 13.12 ng/mL) for the QIAsymphony DSP Circulating DNA Kit.

**FIGURE 4:**
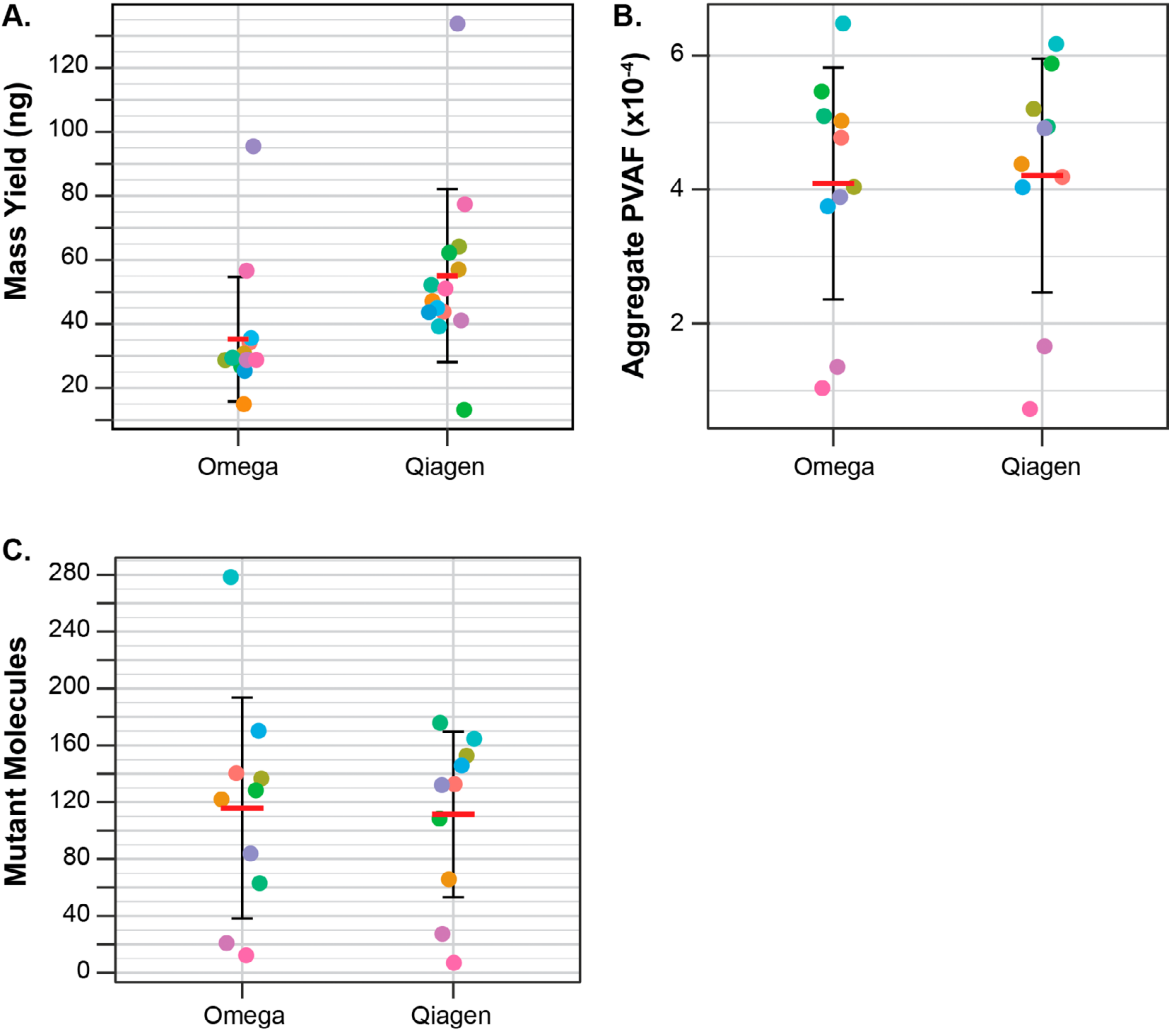
Comparisons of (A) DNA yield, (B) PVAF, and (C) mutant molecules by extraction kit type. Within each graph, the colors indicate individual donors. DNA mass yield, PVAF, and mutant molecules were similar regardless of extraction kit.

After extraction from 10 mL of plasma per donor using each kit, the per‐donor percent cfDNA showed a between‐kit difference ranging from 1.93% to 21.26% (Table 3). For most donors (*n* = 11), this between‐kit difference was less than 11%. The remaining three donors showed differences ranging from 14.72% to 21.26%. On average, the QIAsymphony kit had a 10% higher DNA yield compared to the Omega kit. The Omega kit produced an average DNA yield of 35 ng compared to the average yield of 55 ng from the QIAsymphony kit, both using healthy donor plasma (Figure 4). Conversely, the Omega kit had a higher proportion of cfDNA compared to the QIAsymphony kit. Despite differences in extraction concentrations, mutant molecule and PVAF comparisons suggest that cfDNA extracted from both kits performs similarly (no statistical differences; Figure 4).

**TABLE 3:** cfDNA yield by extraction kit type.

Donor	DLBCL cfDNA spike‐in	Omega kit	QIAsymphony kit	Percent difference (%)
		% cfDNA (40–450 bp)		
1	Yes	73.87	81.1	9.79
2	Yes	68.29	75.14	10.03
3	Yes	72.4	78.16	7.96
4	Yes	62.97	69.24	9.96
5	Yes	78.78	80.3	1.93
6	Yes	76.32	79.38	4.01
7	Yes	69.02	76.1	10.26
8	Yes	56.33	62.22	10.46
9	Yes	72.68	83.38	14.72
10	Yes	80	81.93	2.41
11	Yes	54.78	65.43	19.44
12	Yes	64.5	68.88	6.79
13	No	61.89	75.05	21.26
14	No	62.14	68.71	10.57

### 3.5 DNA Input Mass

All sample replicates across each input mass level (*N* = 60) passed quality control metrics. However, three samples (2 of the 120 ng reference replicates and 1 of the 5 ng replicates) resulted in an MRD negative call with no mutant molecules and a PVAF of 0, despite an expected call of MRD positive, likely due to random sampling of rare mutant molecules in low PVAF samples. These MRD negative result samples were therefore excluded from analysis due to this sampling error, as they were all observed in reference sample replicates near the LoD.

**FIGURE 5:**
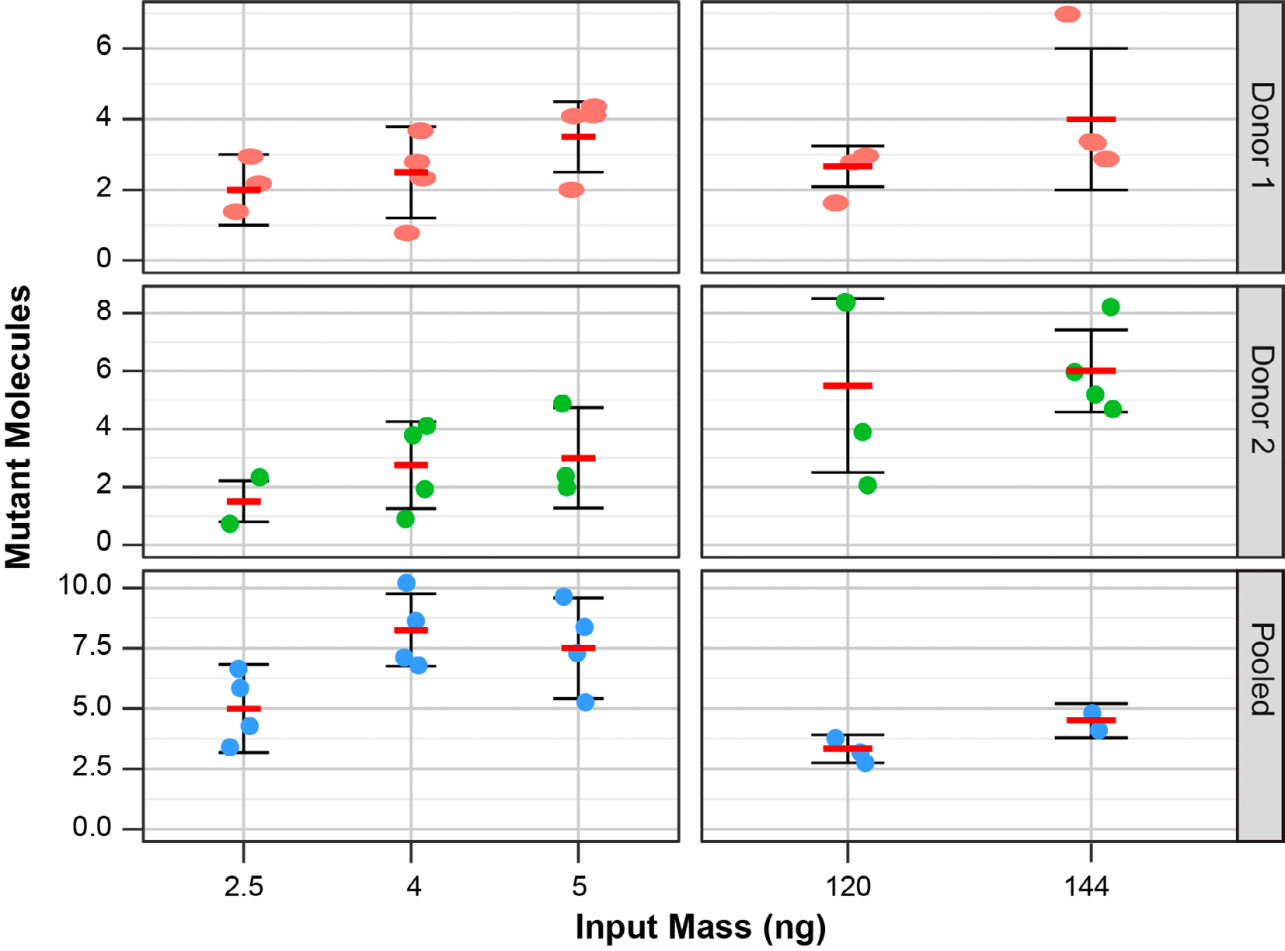
Mutant molecules detected by DNA input mass. Pooled indicates the combination of donors 1 and 2.

For the lower input mass test conditions (2.5 and 4 ng), a total of 88 comparisons were evaluated. The PPA was 77.27% (95% CI 62.16%, 88.53%; 34/44) between the 2.5 ng test and 5 ng reference input mass conditions. The PPA was 100% (95% CI 91.96%, 100%; 44/44) between the 4 ng test and 5 ng reference input mass conditions. The distribution of the mutant molecules for the low test and reference input mass is presented in Figure 5 and shows similar mutant molecules between the 4 ng test (20% below the test minimum) and 5 ng reference.

For the upper input mass test condition (144 ng), a total of 40 comparisons were evaluated. The PPA was 85.00% (95% CI 70.16%, 94.29%; 34/40) between the 144 ng high test and 120 ng reference input mass conditions. The distribution of the mutant molecules for the high test and reference input mass is presented in Figure 5 and shows largely overlapping distributions in mutant molecules detected, indicating no statistically significant difference. The probability of MRD negative calls increases due to a higher number of informative molecules, which requires a corresponding increase in the number of mutant molecules to result in an MRD positive call (Table S7). The MRD‐negative calls were due to replicates containing 0 mutant molecules, likely due to sampling variability between replicates from a sample with a low overall number of mutant molecules.

### 3.6 Reproducibility and Repeatability

Overall ctDNA‐MRD test repeatability and reproducibility APA and ANA were > 99%, with reproducibility across study factors (operators, reagent lots, and instruments) also being > 99% APA and ANA (Table 4). Variability on a per‐sample basis for quantitative measurements across study factors was assessed using mean and CV% of informative molecules (Table S8). CV% ranged from 4.96% to 10.33%. Intercept variance, the proportion of CV% that can be attributed to differences in the baseline level for informative molecules among the groups, ranged from 24.95% to 66.56% (Table S8). Residual variance (3.65%–8.33%) is the portion of variability in the data not accounted for by study factors or intercept variance. Variance from study factors, specifically operator and reagent lot, showed that these factors did contribute to test variability (14.60%–33.27%). Samples from EOT (samples 4 and 5) exhibited higher operator (32.67%–33.27%) and reagent lot variance (32.67%–33.27%) compared to samples 1–3 (14.60%–15.28%). This is primarily due to variations in informative molecules between the smaller number of informative molecules in these EOT samples.

**TABLE 4:** ctDNA‐MRD test repeatability and reproducibility, overall and by study factors.

Metric	Study factor	Agreement	Denominator (from APA or ANA equation)	Numerator (from APA or ANA equation)	Discordant	Agreement
						% (95% CI)
Reproducibility	Overall	APA	10,712.5	10,646	66.5	99.38 (99.21, 99.52)
		ANA	7872	7872	0	100 (99.95, 100)
	Operator	APA	372.5	370	2.5	99.33 (97.87, 99.89)
		ANA	276	276	0	100 (98.67, 100)
	Reagent lot	APA	357	354	3	99.16 (97.56, 99.83)
		ANA	276	276	0	100 (98.67, 100)
	Instrument	APA	462.5	460	2.5	99.46 (98.28, 99.91)
		ANA	276	276	0	100 (98.67, 100)
Repeatability	Overall	APA	180	180	0	100 (95.98, 100)
		ANA	92	92	0	100 (92.29, 100)

## 4 Discussion

Here, we present new data demonstrating that the results of the ctDNA‐MRD test are robust, independent of input sample type, interfering substances, extraction method, and DNA input mass. Taken together with previous analytical validity studies [8], these data show that the PhasED‐Seq‐based ctDNA‐MRD test is a highly reproducible test for assessing residual disease in patients with B‐cell malignancies, with sensitivity robust to various input factors. Test robustness to common sources of variability in laboratory testing is critical, particularly for tests with the potential to inform treatment decisions [6, 17, 18, 19].

The ctDNA‐MRD test requires three sample types to perform MRD testing: somatic/pre‐treatment tumor or plasma, gDNA from WB or PBMCs, and plasma from a subsequent timepoint for MRD monitoring. MRD determination is made by generating a list of tumor‐specific PVs by subtracting any PVs identified in the normal sample from those identified in the somatic sample, and subsequently assessing for the presence of these PVs in the MRD monitoring sample. In this report, we tested alternate sample sources for both the normal and somatic DNA samples. Based on the data presented here, PV list generation is not impacted by using WB compared to PBMCs for the germline sample. Similarly, analytical sensitivity is not impacted by PV lists derived from the different somatic sample sources tested, pre‐treatment plasma or tumor tissue. The limit of detection is approximately three molecules and therefore impacted by Poisson sampling; the differences in total counts of PVs in each list are likely due to stochastic sampling. Both sample types generated a LoD 95% of 3.1 mutant molecules, indicating no significant differences in results based on sample types tested. Based on the data presented here, the number of PVs does not directly impact the LoD; the genomic location of PVs and the total molecules identified using the PV lists are more important than the total count of PVs.

A key step to perform ctDNA‐MRD testing is extraction of cfDNA. A ctDNA‐MRD test is dependent on the presence of mutant molecules in the cfDNA, with ultra‐sensitive methods limited not by analytical sensitivity but by the presence of mutant molecules in the cfDNA. Therefore, methods ensuring optimal recovery of cfDNA molecules from plasma are crucial. Based on the data presented here, DNA yield can be impacted by the specific extraction kit used. However, despite the difference in overall yield, test performance on resultant ctDNA was not impacted, with PVAF and mutant molecule outputs comparable when inputting the same amount of similar quality cfDNA. These data indicate that if a sufficient amount and quality of cfDNA is extracted from a plasma sample, the method used for extraction does not stochastically impact results. This is in line with previous reports comparing cfDNA extraction methods [20, 21, 22].

The ctDNA‐MRD test previously established an input mass range of 5–120 ng [8]. To test the robustness of these input masses, input masses beyond the upper and lower range limits were examined. A decrease in PPA was observed with increased input mass above the upper limit. This is likely because, as greater numbers of informative molecules are detected, the algorithm employs a threshold requiring a higher number of mutant molecules to make an MRD positive call. Sampling variability was also a contributing factor, as the number of mutant molecules present in an individual replicate is variable due to distribution within the sample, and low overall numbers of mutant molecules can result in replicates containing 0 or 1 mutant molecule, resulting in a MRD negative call. An input mass of 4 ng performed similarly to the 5 ng input mass, with performance declining at the 2.5 ng input.

Substances that appear sporadically in patient blood samples have the potential to interfere in laboratory testing processes [23]. Interference is common enough that clinical laboratory methods of validation often examine an excess of these substances in blood to determine if the test will be prone to interference from any of these substances. The common substances to assess for interference include conjugated bilirubin, hemoglobin, and triglycerides. Absolute concentrations of these compounds were 15, 10, and 0.4 μg/μL for triglycerides, hemoglobin, and conjugated bilirubin, respectively, in accordance with CLSI‐EP07 guidelines [24]. The PVAF, informative molecules, and mutant molecules were comparable to controls when testing with the addition of elevated levels of these common interfering substances. There was 100% MRD detection concordance between samples spiked with interfering substances and their controls. Thus, the presence of high levels of these substances did not materially affect test performance under the tested conditions.

Finally, ctDNA‐MRD test variability was assessed. Overall variability in informative molecules ranged from 4.96% to 10.33%, well within the generally accepted ranges of up to 15% allowance [25]. The majority of the observed variance was primarily attributed to sampling variability. Operator and reagent lot did contribute to the overall variability (14.60%–32.67% of %CV), with a larger proportional contribution for samples following therapy as fewer informative molecules were detected. Contribution of instrument and run batch was not directly included as study factors in the model, but their contribution to variability is captured by operator and reagent lot as these are confounding factors.

Overall, the major potential sources of possible variation and interference for the ctDNA‐MRD test were evaluated and not found to have significant impacts on test performance. While there may be additional factors not evaluated in the studies presented here, they are unlikely to have any larger impact than what is shown here, based on test design and execution (residual variance, i.e., variance from all other sources, < 9%). These data further support the analytical performance of the ctDNA‐MRD test and indicate that it is robust to many factors. Combined with previous clinical studies, including recently published studies establishing similar clinical performance in patients with follicular lymphoma [26, 27], this further supports the clinical use of the PhasED‐Seq‐based ctDNA‐MRD test for patients with B‐cell lymphomas.

## Funding

All funding for this work was provided by Foresight Diagnostics Inc.

## Conflicts of Interest

All authors were employed by and received stock options in Foresight Diagnostics Inc. during the design and execution of this study. This work was supported by Foresight Diagnostics Inc.

## Supporting information


**Table S1:** Summary of sample numbers, replicate counts, and experimental conditions across robustness studies.
**Table S2:** Input sample combinations for a single donor.
**Table S3:** PV list size by donor, tumor and germline comparison.
**Table S4:** Chi‐square test results for PVs identified from different germline sample types.
**Table S5:** Impact of interfering substances on performance of the ctDNA‐MRD test.
**Table S6:** Expected PPA for each experimental condition at lower test inputs.
**Table S7:** Probability of MRD ABSENT call by sample and input mass.
**Table S8:** Per‐sample summary statistics and variance analysis.
**Figure S1:** Impact of interfering substances on PV count generation by ctDNA‐MRD test using B‐cell lymphoma cell lines.

## Data Availability

Some data available upon reasonable request to the authors. Not all data will be available for sharing due to proprietary interests.
