# Supplementary material for: Assessment of Potential Sources of Variability on the Analytical Performance of a Circulating Tumor DNA Minimal Residual Disease Test for B‐Cell Lymphomas
Source: J Clin Lab Anal. 2026 May 6;40(11):e70258. doi: 10.1002/jcla.70258 (PMC13267150; doi:10.1002/jcla.70258)
Supplement: Supplementary file 1 — Table S1: Summary of sample numbers, replicate counts, and experimental conditions across robustness studies. Table S2: Input sample combinations for a single donor. Table S3: PV list size by donor, tumor and germline comparison. Table S4: Chi‐square test results for PVs identified from different germline sample types. Table S5: Impact of interfering substances on performance of the ctDNA‐MRD test. Table S6: Expected PPA for each experimental condition at lower test inputs. Table S7: Probability of MRD ABSENT call by sample and input mass. Table S8: Per‐sample summary statistics and variance analysis. Figure S1: Impact of interfering substances on PV count generation by ctDNA‐MRD test using B‐cell lymphoma cell lines. [file JCLA-40-e70258-s001.docx]

# Supplement

## Supplemental Table 1. Summary of Sample Numbers, Replicate Counts, and Experimental Conditions Across Robustness Studies.

| **Study** | **Sample Numbers** | **Replicate Counts** | **Experimental Conditions** |
| --- | --- | --- | --- |
| Sample Source Comparison: Germline DNA | 15 healthy donors | 2 replicates per sample type, 4 total replicates per donor | gDNA extraction from whole blood vs PBMCs |
| Sample Source Comparison: Somatic Plasma or Tumor | 1 DLBCL clinical-contrived sample (MRD positive) | 6 dilution levels, 10 replicates per level | 5 and 120 ng input mass |
| Interfering Substances | 5 (2 contrived samples MRD positive at 2 PVAF levels each and 1 healthy donor sample) | 2 replicates per experimental condition | Reference, Conjugated Bilirubin, Hemoglobin, Triglycerides |
| Extraction Method | 14 healthy donors | 1 replicate per extraction method | 2 extraction methods, donors 1-12 spiked with clinical LBCL cfDNA, Donors 13-14 no spike (healthy donor) |
| DNA Input Mass | 60 samples (3 samples x 4 replicates x 5 levels) | 4 replicates per sample per level | Input Mass levels: 50% below lower input mass (2.5ng), 20% below lower input mass (4ng), lower input mass (5ng), upper input mass (120ng), 20% above upper input mass (144ng) |
| Reproducibility and Repeatability | 3 DLBCL clinical-contrived samples (MRD positive), 2 DLBCL clinical samples (MRD negative) | *DLBCL clinical-contrived samples:* 10-40 replicates per sample/input mass  *DLBCL clinical samples:* 24 replicates per sample/input mass | 5 and 120 ng input mass, 2 reagent lots, 2 operators, 3 instruments, 8 runs |
| Repeatability |  | *DLBCL clinical-contrived samples:* 7 to 24 replicates per specific combination of conditions  *DLBCL clinical samples:* 6 replicates per specific combination of conditions |  |

## Supplemental Table 2. Input Sample Combinations for a Single Donor

| ***Somatic Sample*** | ***Normal Sample*** |
| --- | --- |
| PBMC1 | PBMC2 |
| PBMC1 | WB1 |
| PBMC1 | WB2 |
| PBMC2 | PBMC1 |
| PBMC2 | WB1 |
| PBMC2 | WB2 |
| WB1 | PBMC1 |
| WB1 | PBMC2 |
| WB1 | WB2 |
| WB2 | PBMC1 |
| WB2 | PBMC2 |
| WB2 | WB1 |

PBMC1 and PBMC2 represent replicate samples both extracted from PBMCs isolated from WB, while WB1 and WB2 represent replicate samples extracted directly from WB. By using all possible combinations of these four samples, each donor will have 12 comparisons made for analysis. PBMC, peripheral blood mononuclear cell; WB, whole blood.

## Supplemental Table 3. PV List Size by Donor, Tumor and Germline Comparison

|  |  | **Donor** | | | | | | | | | | | | | | |
| --- | --- | --- | --- | --- | --- | --- | --- | --- | --- | --- | --- | --- | --- | --- | --- | --- |
| **Tumor** | **Germline** | **1** | **2** | **3** | **4** | **5** | **6** | **7** | **8** | **9** | **10** | **11** | **12** | **13** | **14** | **15** |
| PBMC1 | PBMC2 | 0 | 0 | 0 | 0 | 0 | 0 | 0 | 3 | 0 | 0 | 0 | 0 | 0 | 0 | 0 |
| PBMC1 | WB1 | 0 | 0 | 0 | 0 | 0 | 0 | 0 | 3 | 0 | 0 | 0 | 0 | 0 | 0 | 0 |
| PBMC1 | WB2 | 0 | 0 | 0 | 0 | 0 | 0 | 0 | 3 | 0 | 0 | 0 | 0 | 0 | 0 | 0 |
| PBMC2 | PBMC1 | 0 | 1 | 0 | 0 | 0 | 0 | 0 | 0 | 0 | 0 | 0 | 0 | 0 | 0 | 0 |
| PBMC2 | WB1 | 0 | 2 | 0 | 0 | 0 | 0 | 0 | 0 | 0 | 0 | 0 | 0 | 0 | 0 | 0 |
| PBMC2 | WB2 | 0 | 2 | 0 | 0 | 0 | 0 | 0 | 0 | 0 | 0 | 0 | 0 | 0 | 0 | 0 |
| WB1 | PBMC1 | 0 | 0 | 0 | 0 | 0 | 0 | 0 | 0 | 0 | 0 | 0 | 0 | 0 | 0 | 0 |
| WB1 | PBMC2 | 0 | 0 | 0 | 0 | 0 | 0 | 0 | 0 | 0 | 0 | 0 | 0 | 0 | 0 | 0 |
| WB1 | WB2 | 0 | 0 | 0 | 0 | 0 | 0 | 0 | 0 | 0 | 0 | 0 | 0 | 0 | 0 | 0 |
| WB2 | PBMC1 | 0 | 0 | 0 | 0 | 0 | 0 | 0 | 0 | 0 | 0 | 0 | 0 | 0 | 0 | 0 |
| WB2 | PBMC2 | 0 | 0 | 0 | 0 | 0 | 0 | 0 | 0 | 0 | 0 | 0 | 0 | 0 | 0 | 0 |
| WB2 | WB1 | 0 | 0 | 0 | 0 | 0 | 0 | 0 | 0 | 0 | 0 | 0 | 0 | 0 | 0 | 0 |

PV list size indicates differences between PV lists generated by each combination of samples used as the tumor and germline inputs. Comparisons that generated a PV list size >0 are highlighted in red.

## Supplemental Table 4. Chi-square Test Results for PVs Identified from Different Germline Sample Types.

| ***Donor*** | ***Comparison*** | ***Test Statistic*** | ***Degrees of Freedom*** | ***p-Value*** |
| --- | --- | --- | --- | --- |
| 2 | PV/PBMC | 262640 | 262639 | 0.499080 |
| 2 | PV/WB | 262640 | 262639 | 0.499080 |
| 8 | PV/PBMC | 211417 | 211416 | 0.480260 |
| 8 | PV/WB | 211417 | 211416 | 0.479900 |

## Supplemental Table 5. Impact of Interfering Substances on Performance of the ctDNA-MRD Test

| **Cohort** | **Substance** | **PVAF Level** | **N** | **n** | **PPA (95% CI), %** | **NPA (95% CI), %** | **OPA (95% CI), %** |
| --- | --- | --- | --- | --- | --- | --- | --- |
| DB | Conjugated Bilirubin | 2 | 2 | 2 | 100 (15.81, 100) | n/a | n/a |
|  |  | 1 | 2 | 2 | 100 (15.81, 100) |  |  |
|  | Hemoglobin | 2 | 2 | 2 | 100 (15.81, 100) |  |  |
|  |  | 1 | 2 | 2 | 100 (15.81, 100) |  |  |
|  | Triglycerides | 2 | 2 | 2 | 100 (15.81, 100) |  |  |
|  |  | 1 | 2 | 2 | 100 (15.81, 100) |  |  |
| NU-DUL-1 | Conjugated Bilirubin | 2 | 2 | 2 | 100 (15.81, 100) |  |  |
|  |  | 1 | 2 | 2 | 100 (15.81, 100) |  |  |
|  | Hemoglobin | 2 | 2 | 2 | 100 (15.81, 100) |  |  |
|  |  | 1 | 2 | 2 | 100 (15.81, 100) |  |  |
|  | Triglycerides | 2 | 2 | 2 | 100 (15.81, 100) |  |  |
|  |  | 1 | 2 | 2 | 100 (15.81, 100) |  |  |
| Combined  DB + NU-DUL-1 | Conjugated Bilirubin | 1 and 2 | 8 | 8 | 100 (63.30, 100) |  |  |
|  | Hemoglobin | 1 and 2 | 8 | 8 | 100 (63.30, 100) |  |  |
|  | Triglycerides | 1 and 2 | 8 | 8 | 100 (63.30, 100) |  |  |
| Healthy Donor | Conjugated Bilirubin | n/a | 4 | 4 | n/a | 100 (39.76, 100) |  |
|  | Hemoglobin |  | 4 | 4 |  | 100 (39.76, 100) |  |
|  | Triglycerides |  | 4 | 4 |  | 100 (39.76, 100) |  |
| Overall | Conjugated Bilirubin | n/a | 12 | 12 | n/a | | 100 (73.54, 100) |
|  | Hemoglobin |  | 12 | 12 |  |  | 100 (73.54, 100) |
|  | Triglycerides |  | 12 | 12 |  |  | 100 (73.54, 100) |

NPA, negative percent agreement; OPA, overall percent agreement; PPA, positive percent agreement; PVAF, phased variant allele fraction.

## Supplemental Table 6. Expected PPA for Each Experimental Condition at Lower Test Inputs

| **Interfering Substance** | ***Input Mass (ng)**** | ***PPA (95% CI)*** |
| --- | --- | --- |
| Control | 15 | 99.9 (100 - 100) |
|  | 7.5 | 99.2 (87.5 - 100) |
|  | 5 | 98.1 (87.5 - 100) |
| Conjugated Bilirubin | 15 | 99.8 (100 - 100) |
|  | 7.5 | 98.7 (87.5 - 100) |
|  | 5 | 97.0 (87.5 - 100) |
| Hemoglobin | 15 | 100 (100 - 100) |
|  | 7.5 | 99.9 (100 - 100) |
|  | 5 | 99.5 (87.5 - 100) |
| Triglycerides | 15 | 100 (100 - 100) |
|  | 7.5 | 99.6 (87.5 - 100) |
|  | 5 | 98.4 (87.5 - 100) |

*As the input mass decreases the relative number of informative molecules is similarly decreased. For example, when decrease input mass from 15 ng to 7.5 ng, the number of informative molecules is also halved. An input mass of 5 ng would be expected to have 1/3 of the informative molecules compared to the 15 ng input. CI, confidence interval; ng, nanogram; PPA, positive percent agreement.

## Supplemental Figure 1. Impact of Interfering Substances on PV Count Generation by ctDNA-MRD Test Using B-Cell Lymphoma Cell Lines


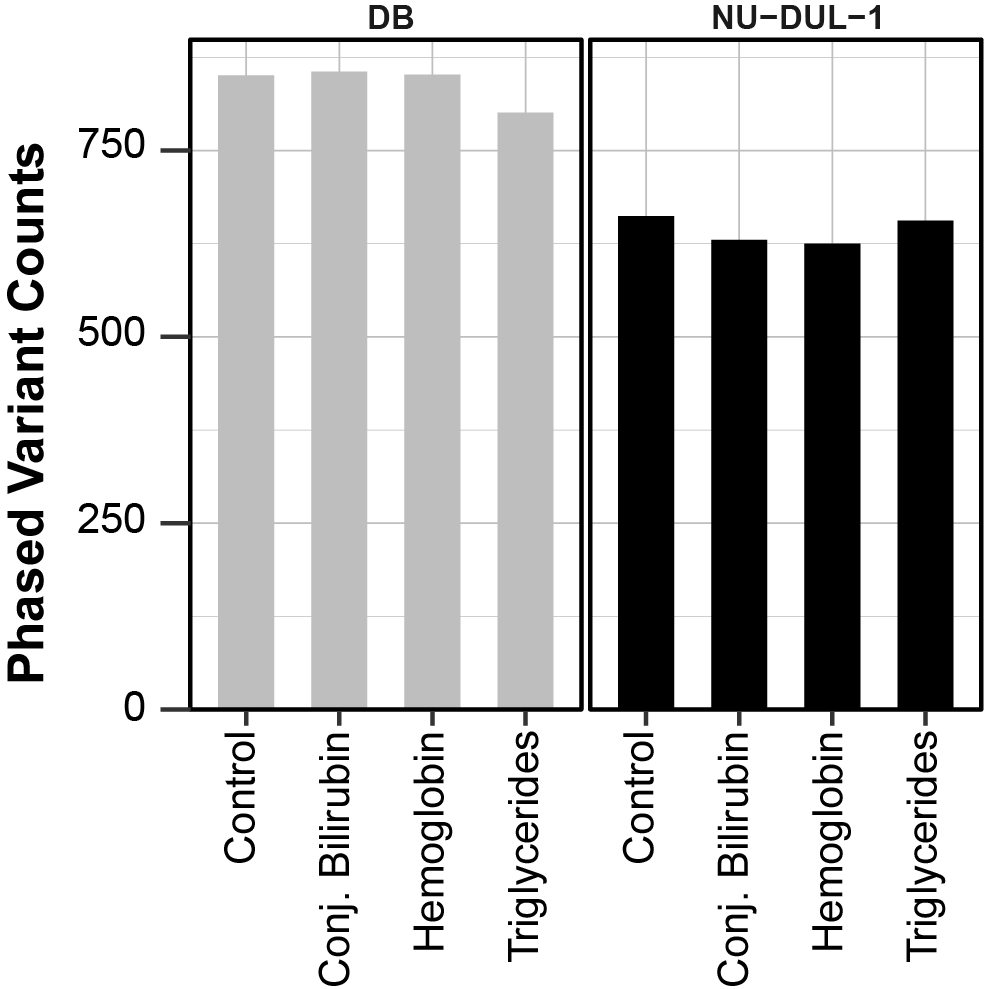


## Supplemental Table 7. Probability of MRD ABSENT Call by Sample and Input Mass

Probability of MRD Absent Call was calculated by using the Poisson distribution based on the average number of mutant molecules detected and the number of mutant molecules required to make an MRD present call. Each row indicated a sample replicate, with 4 replicates per donor per input mass.

| ***Donor*** | ***Input Mass (ng)*** | ***Mutant Molecules*** | ***Informative Molecules*** | ***MRD Call*** | ***Average MM Detected*** | ***Probability of MRD Absent Call (%)*** |
| --- | --- | --- | --- | --- | --- | --- |
| 1 | 2.5 | 0 | 17,936 | ABSENT | 1.50 | 22.31 |
| 1 | 2.5 | 3 | 19,732 | PRESENT | 1.50 | 22.31 |
| 1 | 2.5 | 1 | 17,847 | PRESENT | 1.50 | 22.31 |
| 1 | 2.5 | 2 | 18,360 | PRESENT | 1.50 | 22.31 |
| 1 | 4 | 1 | 27,221 | PRESENT | 2.50 | 8.21 |
| 1 | 4 | 2 | 25,563 | PRESENT | 2.50 | 8.21 |
| 1 | 4 | 4 | 26,973 | PRESENT | 2.50 | 8.21 |
| 1 | 4 | 3 | 26,328 | PRESENT | 2.50 | 8.21 |
| 1 | 5 | 4 | 34,333 | PRESENT | 3.50 | 3.02 |
| 1 | 5 | 2 | 34,111 | PRESENT | 3.50 | 3.02 |
| 1 | 5 | 4 | 33,898 | PRESENT | 3.50 | 3.02 |
| 1 | 5 | 4 | 33,631 | PRESENT | 3.50 | 3.02 |
| 1 | 120 | 2 | 463,169 | PRESENT | 2.00 | 13.53 |
| 1 | 120 | 0 | 462,273 | ABSENT | 2.00 | 13.53 |
| 1 | 120 | 3 | 466,148 | PRESENT | 2.00 | 13.53 |
| 1 | 120 | 3 | 454,250 | PRESENT | 2.00 | 13.53 |
| 1 | 144 | 3 | 646,503 | PRESENT | 4.00 | 1.83 |
| 1 | 144 | 3 | 699,411 | PRESENT | 4.00 | 1.83 |
| 1 | 144 | 7 | 680,426 | PRESENT | 4.00 | 1.83 |
| 1 | 144 | 3 | 661,069 | PRESENT | 4.00 | 1.83 |
| 2 | 2.5 | 1 | 49,899 | PRESENT | 0.75 | 47.24 |
| 2 | 2.5 | 0 | 49,061 | ABSENT | 0.75 | 47.24 |
| 2 | 2.5 | 0 | 49,660 | ABSENT | 0.75 | 47.24 |
| 2 | 2.5 | 2 | 50,009 | PRESENT | 0.75 | 47.24 |
| 2 | 4 | 4 | 75,824 | PRESENT | 2.75 | 6.39 |
| 2 | 4 | 2 | 74,461 | PRESENT | 2.75 | 6.39 |
| 2 | 4 | 1 | 75,781 | PRESENT | 2.75 | 6.39 |
| 2 | 4 | 4 | 75,942 | PRESENT | 2.75 | 6.39 |
| 2 | 5 | 0 | 100,818 | ABSENT | 2.25 | 10.54 |
| 2 | 5 | 2 | 99,577 | PRESENT | 2.25 | 10.54 |
| 2 | 5 | 5 | 97,022 | PRESENT | 2.25 | 10.54 |
| 2 | 5 | 2 | 101,876 | PRESENT | 2.25 | 10.54 |
| 2 | 120 | 8 | 1,753,651 | PRESENT | 5.50 | 2.66 |
| 2 | 120 | 2 | 1,712,704 | PRESENT | 5.50 | 2.66 |
| 2 | 120 | 4 | 1,675,453 | PRESENT | 5.50 | 2.66 |
| 2 | 120 | 8 | 1,703,625 | PRESENT | 5.50 | 2.66 |
| 2 | 144 | 8 | 2,033,891 | PRESENT | 6.00 | 1.74 |
| 2 | 144 | 6 | 2,026,841 | PRESENT | 6.00 | 1.74 |
| 2 | 144 | 5 | 2,019,635 | PRESENT | 6.00 | 1.74 |
| 2 | 144 | 5 | 2,007,257 | PRESENT | 6.00 | 1.74 |
| Pooled | 2.5 | 7 | 111,846 | PRESENT | 5.00 | 0.67 |
| Pooled | 2.5 | 6 | 113,239 | PRESENT | 5.00 | 0.67 |
| Pooled | 2.5 | 3 | 110,915 | PRESENT | 5.00 | 0.67 |
| Pooled | 2.5 | 4 | 114,933 | PRESENT | 5.00 | 0.67 |
| Pooled | 4 | 7 | 202,892 | PRESENT | 8.25 | 0.03 |
| Pooled | 4 | 7 | 204,347 | PRESENT | 8.25 | 0.03 |
| Pooled | 4 | 9 | 199,128 | PRESENT | 8.25 | 0.03 |
| Pooled | 4 | 10 | 204,578 | PRESENT | 8.25 | 0.03 |
| Pooled | 5 | 7 | 247,987 | PRESENT | 7.5 | 0.06 |
| Pooled | 5 | 8 | 236,168 | PRESENT | 7.5 | 0.06 |
| Pooled | 5 | 5 | 235,596 | PRESENT | 7.5 | 0.06 |
| Pooled | 5 | 10 | 222,950 | PRESENT | 7.5 | 0.06 |
| Pooled | 120 | 4 | 3,782,095 | PRESENT | 2.5 | 28.73 |
| Pooled | 120 | 3 | 3,916,222 | PRESENT | 2.5 | 28.73 |
| Pooled | 120 | 3 | 3,940,440 | PRESENT | 2.5 | 28.73 |
| Pooled | 120 | 0 | 3,860,687 | ABSENT | 2.5 | 28.73 |
| Pooled | 144 | 1 | 4,566,542 | ABSENT | 2.75 | 23.97 |
| Pooled | 144 | 1 | 4,480,156 | ABSENT | 2.75 | 23.97 |
| Pooled | 144 | 5 | 4,728,462 | PRESENT | 2.75 | 48.15 |
| Pooled | 144 | 4 | 4,640,960 | PRESENT | 2.75 | 48.15 |

##

## Supplemental Table 8. Per-Sample Summary Statistics and Variance Analysis

| **Sample** | **Input Mass (ng)** | **Mean IM** | **SD** | **CV%** | **Intercept Variance** | **Residual Variance** | **Operator Variance** | **Reagent Lot Variance** |
| --- | --- | --- | --- | --- | --- | --- | --- | --- |
|  |  |  |  |  | **% of CV%** | | | |
| 1 – MRD Positive | 5 | 225,735.60 | 15655.55 | 6.94 | 66.54 | 3.66 | 15.25 | 14.60 |
|  | 120 | 4,010,029.30 | 307746.34 | 7.67 | 66.33 | 3.67 | 15.24 | 14.63 |
| 2 – MRD Positive | 5 | 95,678.40 | 6056.32 | 6.33 | 66.45 | 3.65 | 15.27 | 14.65 |
|  | 120 | 1,687,422.00 | 111880.52 | 6.63 | 66.56 | 3.65 | 15.25 | 14.63 |
| 3 – MRD Positive | 5 | 31,487.55 | 2440.54 | 7.75 | 66.44 | 3.66 | 15.28 | 14.60 |
|  | 120 | 604,428.15 | 62440.96 | 10.33 | 66.51 | 3.66 | 15.20 | 14.63 |
| 4 – MRD Negative | 5 | 15,215.21 | 818.34 | 5.38 | 26.65 | 8.10 | 32.67 | 32.67 |
| 5 – MRD Negative | 5 | 24,293.08 | 1204.32 | 4.96 | 24.95 | 8.33 | 33.27 | 33.27 |

IM, Informative Molecules; SD, standard deviation; CV%, percent coefficient of variance
